# Machine Learning with Iterative Error-Driven Refinement for Predicting PFAS-Coated Surface Wettability

**DOI:** 10.1021/acsmaterialsau.6c00228

**Published:** 2026-10-24

**Authors:** Yilang Liu, Jiaqi Yang, Bahareh Rahimi, Tejasri Remma, Joshna Chittigori, Jayant Kumar, Gabriel Salierno, Margaret A. Auerbach, Ravi Mosurkal, Ramaswamy Nagarajan, Fanglin Che

**Affiliations:** † Department of Chemical Engineering, 542588 University of Massachusetts Lowell, Lowell, Massachusetts 01854, United States; ‡ Department of Chemical Engineering, Worcester Polytechnic Institute, Worcester, Massachusetts 01609, United States; § Department of Plastics Engineering, 14710 University of Massachusetts Lowell, Lowell, Massachusetts 01854, United States; ∥ Department of Physics, University of Massachusetts Lowell, Lowell, Massachusetts 01854, United States; ⊥ Toxics Use Reduction Institute (TURI), University of Massachusetts Lowell, Lowell, Massachusetts 01854, United States; # Soldier Protection Directorate, US Army DEVCOM Soldier Center, Natick, Massachusetts 01760, United States

**Keywords:** Intrinsic Water and Oil Wettability, PFAS Coating, Molecular Dynamics, Machine Learning, Water and Oil Contact Angle Prediction

## Abstract

PFAS coatings impart strong water and oil repellency on materials, but their environmental persistence necessitates sustainable alternatives. The development of alternative chemistries is hindered by limited mechanistic understanding of how molecular structure and surface organization govern intrinsic water and oil wettability. Here, a machine learning-driven molecular dynamics (MD) simulation framework was developed to predict and rationalize the intrinsic water and oil wettability of PFAS coatings on nylon for both water and oil. By systematically sampling PFAS head group types (−CHO, −COOH, −OH, and −SO_3_H), chain lengths from C1–C7, surface packing densities (1.42–4.65 nm^–2^), and both linear and branched isomers, a comprehensive database of MD-derived contact angles was constructed. A data-efficient, error-driven refinement strategy enables accurate prediction of water and oil contact angles with mean absolute errors below 3° for linear PFAS and below 5° for branched isomers. Analysis shows that intrinsic water and oil repellency peaks at intermediate packing densities rather than at the highest packing density. Moreover, water repellency depends more on chain length than oil repellency, and head group chemistry strongly modulates these effects. Oil contact angles are typically ∼10–20° lower than water contact angles, consistent with experimental observations. This work provides molecular-level insight into the intrinsic water and oil wettability of PFAS coatings and establishes a data-driven framework that may support the future rational design and screening of environmentally benign, non-PFAS coating chemistries.

## Graphical abstract

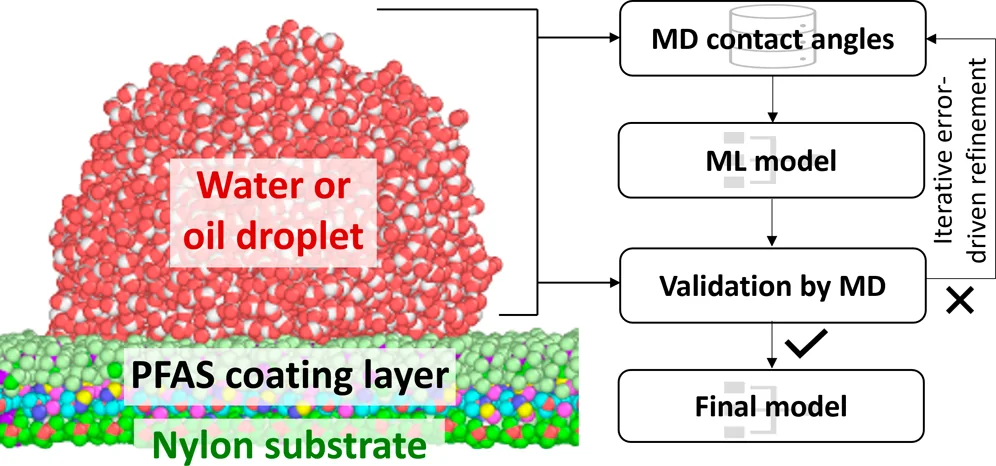

## 1 Introduction

Per- and polyfluoroalkyl substances (PFAS) are a class of chemicals widely known for their strong resistance to liquids.^1^ This behavior originates from fluorocarbon (CF_*x*_) groups, which possess low polarizability and therefore impart low surface energy and weak intermolecular interactions with liquids, while the strong C–F bond contributes to the chemical stability of PFAS.^2,3^ As a result, PFAS-coated materials exhibit durable hydrophobicity and, in many cases, oleophobicity, giving rise to omniphobic behavior that is difficult to achieve using conventional hydrocarbon-based coatings.^4^ These attributes have led to the extensive use of PFAS in textile applications, including protective clothing, where long-lasting resistance to water and oil under extreme environmental conditions is required.

PFAS are highly persistent chemicals in the environment, with a tendency to bioaccumulate in living organisms, and have a high resistance to natural degradation processes, as recognized in the scientific literature and by various regulatory sources.^5,6^ Growing pressure has therefore intensified efforts to develop environmentally benign, non-PFAS alternatives.^7,8^ Despite substantial interest, progress toward viable non-PFAS omniphobic coatings has been limited. While several non-PFAS finishes can achieve water repellency, they generally fail to provide the combination of oil repellency and durability necessary for demanding textile applications at low loading levels.^7,9–12^

A major challenge in developing effective alternatives is the lack of a mechanistic understanding of how molecular features of PFAS, such as fluorine content, chain length, branching, head group chemistry, and surface packing, govern macroscopic liquid repellency on textile substrates. In the absence of well-defined structure–property relationships, the design of chemistry-based PFAS alternatives has largely relied on empirical trial-and-error approaches, limiting predictive capability and transferability.

To build structure–property relationships, liquid repellency of coating materials has traditionally been investigated through experimental approaches. The intrinsic water and oil wettability of planar surfaces is commonly characterized using the equilibrium contact angle of water and oil droplets, respectively, as described by Young’s equation.^13–16^ In the present study, equilibrium contact angles are used as descriptors of the intrinsic wettability of the modeled planar PFAS-coated surfaces. Dynamic wetting characteristics, including contact angle hysteresis, roll-off angle, and droplet mobility, are not evaluated and are beyond the scope of the present study. For PFAS coatings, however, contact angles are more complex because they are influenced by the large number of variations of PFAS chemical structures of the head group and the carbon chain length, in addition to their packing density.^17,18^ Understanding the effects of these variables is essential for establishing molecular-level structure–property relationships. These factors obscure intrinsic interfacial properties and hinder systematic comparison across different coating chemistries, rendering experiments alone insufficient for establishing structure–property correlations.

These limitations motivate the use of computational methods as a complementary tool.^19–21^ Molecular dynamics (MD) simulations enable controlled investigation of interfacial phenomena at the molecular scale and provide access to intrinsic wettability through simulated contact angles. Numerous theoretical studies have been conducted to elucidate surface hydrophobicity.^18,22–25^ For example, Yadav et al. studied hydrophobicity using MD simulations of self-assembled perfluoroalkyl acrylate (PFA) surfaces, examining the effects of PFA packing density and chain length on the water contact angle.^18^ They observed that hydrophobicity is sensitive at low packing density and short PFA chains. Specifically, water contact angles increase with packing density in PFA with a chain length of two, whereas longer PFA chains exhibit similar hydrophobicity across different packing densities from 1.95 to 4.63 nm^–2^. In terms of chain length effects, surface hydrophobicity increases with increasing PFA chain length at lower packing densities once the chain exceeds six carbon atoms, while at higher packing densities it slightly decreases as chain length increases.

However, prior experimental and computational studies have largely focused on simplified PFAS coating chemistries, including single-chain PFAS, with primary emphasis on intrinsic water wettability rather than the apparent contact angles influenced by textiles.^18^ As a result, textile-relevant architectures, branched PFAS structures, and oil repellency have been largely overlooked. In practical textile systems, the apparent contact angle is governed not only by the intrinsic wettability of the coating material, but also by the coupled effects of coating chemistry, molecular organization, surface roughness, and textile morphology.^26^ Consequently, applying PFAS coatings to textile substrates can substantially modify both water and oil contact angles. However, existing databases and modeling efforts lack systematic information capturing the combined influence of PFAS coating structure and textile morphology on both water and oil repellency, thus limiting their utility for predictive non-PFAS coating material design/discovery.^4,27,28^

PFAS comprise a broad class of molecules with diverse structures that vary in chain length and isomeric form. When PFAS are applied as coatings on substrates, an additional degree of variability arises from differences in packing density.^18^ Together, these factors create a vast configurational space that is impractical to explore exhaustively through experiments alone. Even MD simulations become prohibitively expensive when attempting to comprehensively sample this space. Consequently, there is a critical need for predictive, physically grounded modeling frameworks that can efficiently explore this space and identify the molecular features most responsible for omniphobic behavior.

Machine learning (ML) approaches have successfully accelerated materials discovery in other domains and offer a promising pathway for identifying the key chemical and physical characteristics required to replicate PFAS properties.^29–34^ The present study utilizes a machine learning algorithm with an error-driven refinement framework to predict the intrinsic water and oil wettability behavior of PFAS coatings toward both water and oil obtained using MD simulations. This analysis considers four common head groups (−CHO, −COOH, −OH, and −SO_3_H), chain lengths ranging from 1 to 7, and surface packing densities spanning 1.42 to 4.65 nm^–2^. The resulting ML model predicts water and oil contact angles with a mean absolute error (MAE) below 3°. In addition, this study systematically analyzes the effects of head group types, chain length, and packing density on liquid repellency. The insights gained from this study provide a mechanistic understanding of PFAS-based intrinsic water and oil wettability and establish a foundation for the rational design of durable non-PFAS alternatives by leveraging the relationships between chain length, head group chemistry, and packing density identified in this work.

## 2 Methods

### 2.1 Molecular Dynamics (MD) Simulation Setup

The PFAS and Nylon substrate were modeled using the all-atom Optimized Potentials for Liquid Simulations (OPLS) force field,^35,36^ while water molecules were modeled using the extended simple point charge (SPC/E) model^37^ and *n*-decane molecules were described using a united atom OPLS force field.^38^ Nylon was selected as a chemically relevant polymer substrate for systematically investigating PFAS coating chemistry under well-controlled atomistic conditions. Both short-range Lennard-Jones interactions and Coulombic interactions were truncated at 1.0 nm. The system was simulated for 1 ns under a constant number of particles, volume, and temperature (canonical NVT) ensemble at 300 K, using a Nosé-Hoover thermostat to reach equilibrium. All MD simulations were performed within Large-scale Atomic/Molecular Massively Parallel Simulator (LAMMPS) package.^39^

To facilitate high-throughput MD simulations, an automated workflow was developed to generate all initial structures for the oil/water-PFAS-Nylon systems, covering all considered PFAS and their packing densities (*d*_p_) on the Nylon substrate. Initially, all unique PFAS molecules with four different head groups and chain lengths ranging from 1 to 7 were enumerated using the Simplified Molecular Input Line Entry System (SMILES).^40^ Given SMILES, the structure of each PFAS molecule was generated and optimized using universal force field^41^ within RDKit Python package^42^ to obtain the coordinates of the atoms of the PFAS molecule. The coordinates were then rotated to evaluate the head group at a lower location, attaching to Nylon and CF_*x*_ groups facing up to interact with oil/water molecules above it, ensuring consistency with a real wetting process.

We selected a Nylon substrate to support the PFAS chains and prevent them from orienting in the opposite direction. The Nylon substrate was selected as a chemically and military relevant polymer substrate that enables systematic investigation of PFAS coating chemistry under well-controlled molecular conditions. The present study focuses on PFAS coatings on an idealized flat Nylon surface as a tractable model system for establishing molecular-level structure–property relationships. Real textile wettability is considerably more complex because it depends not only on coating chemistry but also on fiber morphology, weave architecture, surface roughness, porosity, coating uniformity, liquid penetration, and multiscale capillary effects.^43^ These structural and morphological factors govern the apparent wettability of coated fabrics and are beyond the scope of the present MD simulations. Therefore, the present work should be viewed as a first step toward understanding the intrinsic wetting behavior of PFAS coatings. Future work will integrate these molecular insights with mesoscale or continuum descriptions of textile morphology to predict the apparent wetting behavior of coated fabrics.

A one-layer Nylon-6,6 slab model exposing a (010) facet was employed to represent the Nylon surface, ensuring strong bonding between polyamide chains.^44,45^ The periodic cell of Nylon-6,6 was repeated by 4 and 16 times, forming a supercell with a size of 11.76 and 10.07 nm along x and y axes, respectively, and an angle of 86.48 degrees between the two axes. A 200 nm empty space was created to accommodate PFAS coating and a liquid droplet, avoiding interaction with atoms in the adjacent periodic supercell. PFAS molecules were stacked evenly along x and y axes by specific counts to construct the systems of a specific *d*_p_ (Table 1). The head groups of PFAS molecules are ∼0.1 nm above Nylon, ensuring the attachment between PFAS and Nylon. The PFAS head groups were terminated with a carbon atom to represent their bond formation with the substrate, similar to a previous MD study for water contact angle on perfluoroalkyl acrylate coating.^18^ A common attachment geometry was used for all PFAS head group chemistries to enable controlled comparison of head group identity, fluorinated chain length, and packing density. This idealized representation does not explicitly capture all possible bonding configurations, hydrogen bonding environments, or interfacial orientations that may occur experimentally and should therefore be interpreted as a comparative molecular model rather than a detailed reconstruction of a specific PFAS-Nylon interface.

**Table 1:** Arrangement of PFAS Molecules and the Resulting Packing Density

Number of PFAS along *x* axis	Number of PFAS along *y* axis	Packing density, *d*_p_ (nm^–2^)
12	14	1.42
15	17	2.16
18	21	3.20
20	23	3.89
22	25	4.65

The oil and water droplets were represented using 300 *n*-decane molecules and 3000 water molecules, respectively. Such droplet sizes have been found giving similar contact angle compared to larger droplet sizes and standard errors lower than 5° as shown in Figure S2 and Tables S1 and S2. Grade 4-8 oil repellency is required for many fabrics used in defense applications, where resistance to hydrocarbons is critical. According to the AATCC TM118 standard, grade 6 oil repellency indicates resistance to *n*-decane, which has a surface tension of approximately 23.8 mN/m.^46^ A droplet was placed above the PFAS molecules by around 0.1 nm in the geometry model. Simulations comprised five *d*_p_ ranging from 1.42 to 4.65 nm^–2^ by varying the number of PFAS molecules along the x- and *y*-axes, as summarized in Table 1. This *d*_p_ range has previously been used for water contact angle derivation from MD simulation for coating of perfluoroalkyl acrylate with chain length from 2 to 10, exhibiting good agreement with experimental results.^18^ An illustration of the geometry model for the water-PFAS-Nylon system at a *d*_p_ of 3.20 nm^–2^ is shown in Figure 1.

**Figure 1:**
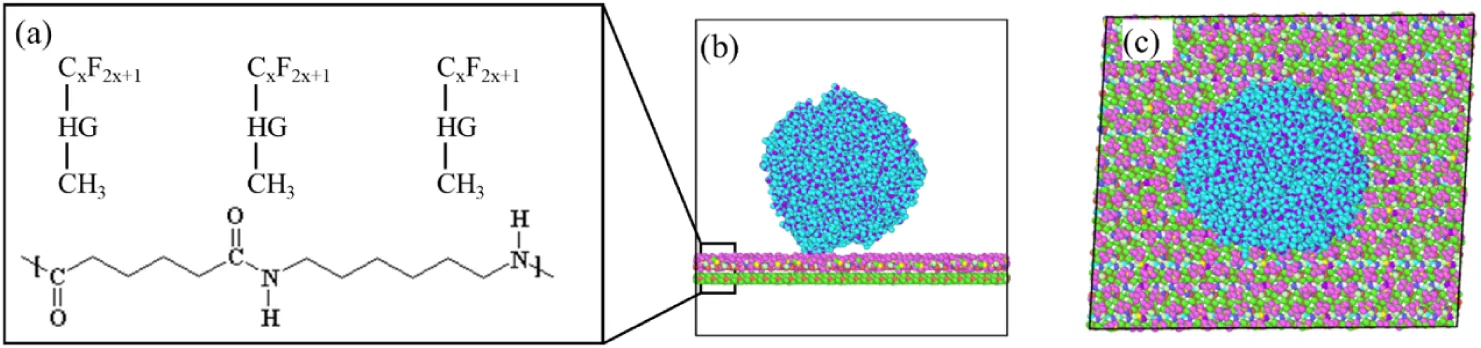
Molecular models of the PFAS-coated Nylon substrate and initial wetting configuration. (a) Zoom-in molecular structure of PFAS-Nylon, where the head group (HG) is terminated by a CH_3_. (b) Side view and (c) top view of initial structure of a water droplet on the PFAS coated Nylon slab model, (010) facet. The blue, purple, and green ensembles represent water droplet, PFAS coating layer, and Nylon(010) substrate, respectively.

### 2.2 Contact Angle Derivation from Molecular Dynamics Trajectory

The contact angle of a liquid droplet on PFAS coating was derived by fitting the equilibrated droplet to a spherical shape. First, the droplet center of the *x*–*y* plane was obtained as the average x and y coordinates of all molecules of water or *n*-decane molecules. The droplet was then sectioned into 1 Å^3^ cubes, and the density of each section was calculated by averaging the nearby 4 Å^3^ spatial binning. The radius (*R*) of each section to the center of the *x*–*y* plane was calculated using the coordinates. For every identical pair of R and height (Z), the density was averaged. With these derivations, spatial density profile was obtained as a function of R and Z, with an example illustrated in Figure S1. The water–air interface was identified for sections with densities within 400 to 600 kg/m^3^, and these interface coordinates were used for fitting to a circle equation. The baseline height was calculated as the average of the *z*-direction coordinates of the interface between water and PFAS coating, which was also determined when the densities are within 400 to 600 kg/m^3^. The contact angle of water droplet was found at the intersection of the fitted circle and PFAS coating height. Similarly, *n*-decane-air interface was identified at the divided sections with densities within 265 to 465 kg/m^3^ to obtain *n*-decane contact angles. We tested different density ranges determining the interface and spatial binning and found the results are not sensitive to the density ranges and spatial binning (Figures S4–S7). The final water and *n*-decane contact angles were obtained by averaging the contact angles over 250–500 ps for water and 750–1000 ps for *n*-decane, respectively, after the values reached stable plateaus (Figure S3).

### 2.3 Experimental Methods

#### 2.3.1 PFAS Coating and ODA Coating Synthesis

The PFAS-treated control sample was a plain-weave Nylon-6,6 fabric supplied by Brookwood Companies Inc. andcommercially treated with a C6 fluorochemical finish, which is commonly used in textile finishing to impart water and oil repellency. The fabric had a weight of 3.5 oz/y^2^ and a minimum of 157 warp yarns and 66 filling yarns per inch. It was used as received, without further surface modification, and was selected as an experimental benchmark representative of current PFAS-treated Nylon fabrics.

The octadecylamine­(ODA)-coated Nylon-6,6 sheet was prepared using a two-step surface modification process with polydopamine (PDA) as a primer layer. Nylon sheets were used as model substrates to provide a smooth and well-defined surface that better matched the modeling system. First, the Nylon sheet was immersed in an alkaline Tris base solution containing dopamine hydrochloride at pH 8.5 and stirred for 12 h to allow dopamine self-polymerization and PDA deposition on the surface. The PDA-coated sheet was then removed, thoroughly rinsed with deionized water, and dried at 60 °C. Next, ODA, at 20 wt % relative to the substrate weight, was dissolved in ethanol and applied to the PDA-coated Nylon sheet through drop coating. After treatment, the sample was washed with ethanol to remove excess and unbound ODA, then oven-dried at 80 °C for 2 h to remove residual solvent and improve coating adhesion. The resulting ODA-treated Nylon sheet was used for water and oil repellency evaluation.

#### 2.3.2 Contact Angle Measurement

Water and oil repellency were evaluated for both the PFAS-treated fabrics and Nylon sheets treated with PFAS-free formulations. The Biolin Scientific Attension Theta Flex apparatus (Sweden), following ASTM D5946, was employed to measure contact angles. The liquids tested were deionized water (surface tension ≈ 72.0 mN/m) and n-decane (surface tension ≈ 23.8 mN/m), which corresponds to Grade 6 of the AATCC TM118 oil repellency test and was used here only as a probe liquid for contact angle measurements.^46^ The AATCC TM 118 grading test was not performed, since its visual grades cannot be compared directly with the contact angles obtained from MD simulations. Each measurement was repeated five times at different spots on the sample surface to ensure consistency.

## 3 Results and Discussion

### 3.1 Repellency to Water and Oil on Single-Chain PFAS Coating

Using the established high-throughput simulation framework and MD-based contact-angle analysis, the water- and oil-repellency of single-chain PFAS coatings on Nylon is expressed as a function of molecular structure and surface packing density. Contact angles were evaluated for both water and *n*-decane droplets, and the results at the lowest and highest packing densities (*d*_p_) are presented as functions of chain lengths in Figures 2–3, respectively, with results for intermediate *d*_p_ values provided in the Supplementary Data. The assessment of the contact angles of water and *n*-decane derived from our MD simulations is presented in Section 3.4. We benchmarked the MD-derived contact angles against experimental measurements and literature values reported for PFAS coatings. In addition, we further benchmarked the MD workflow using octadecylamine (ODA)-coated Nylon sheets, to assess the reliability of the simulated contact-angle predictions. The assessed MD data set is subsequently used to train the machine-learning model, which serves as a computational surrogate for the MD-derived response surface within the simulated design space and is not intended to provide independent experimental ground truth.

**Figure 2:**
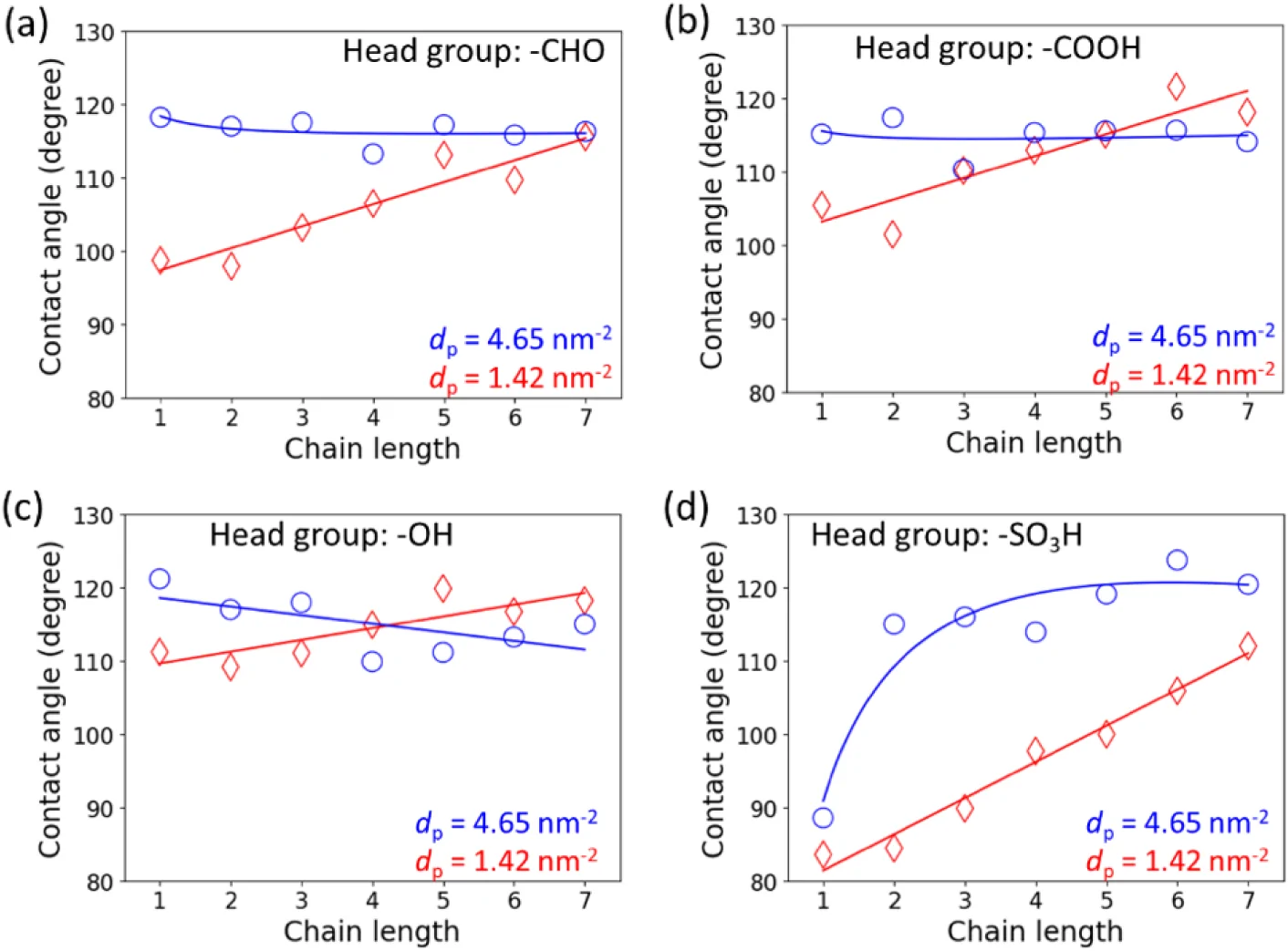
Contact angle of water droplet on Nylon coated by single chain PFAS with head group (a) −CHO, (b) −COOH, (c) −OH, and (d) −SO_3_H, as a function of PFAS chain length, at packing densities of 1.42 nm^–2^ (red) and 4.65 nm^–2^ (blue).

**Figure 3:**
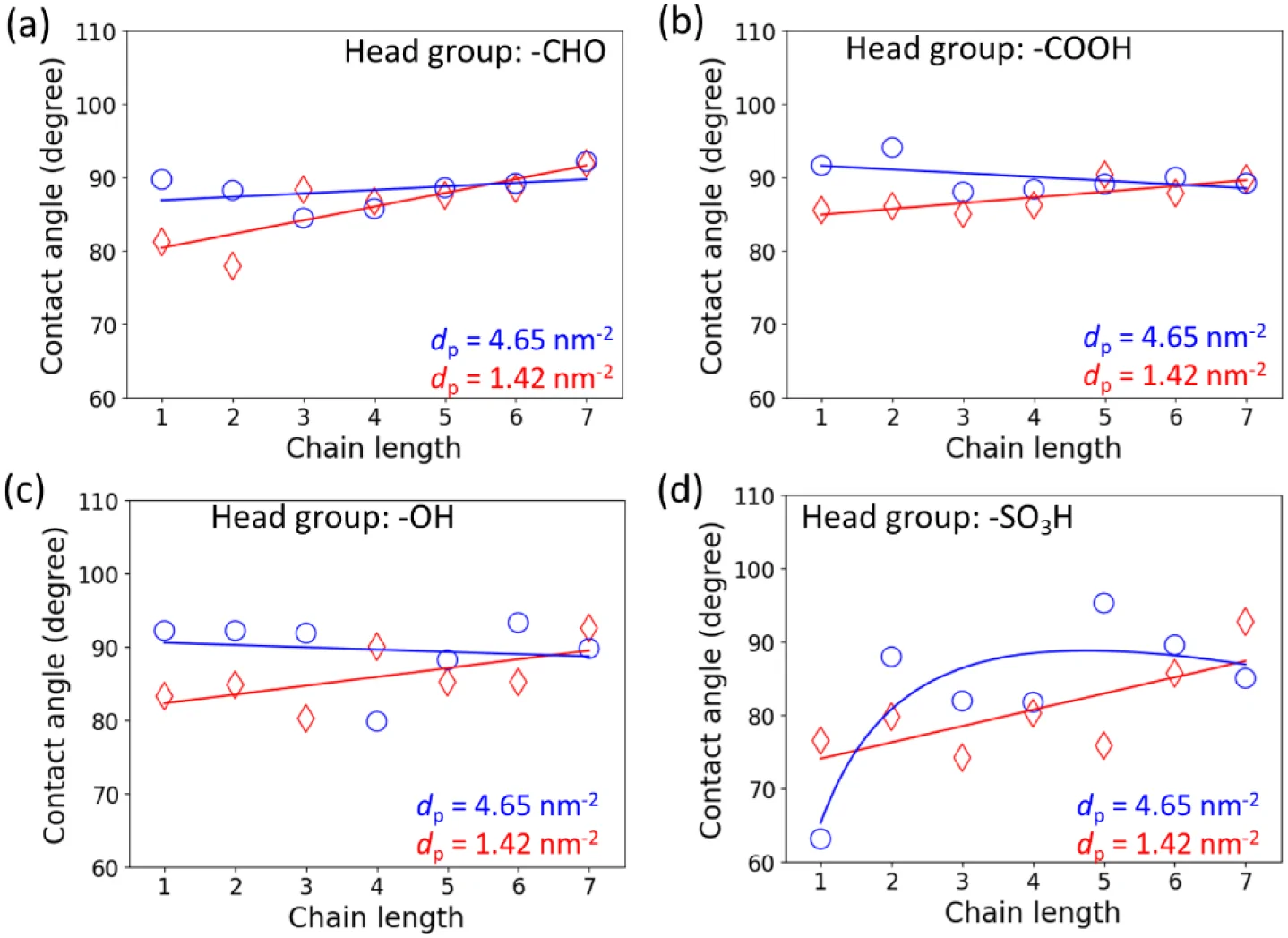
Contact angle of oil droplet on Nylon coated by single chain PFAS with head group (a) −CHO, (b) −COOH, (c) −OH, and (d) −SO_3_H, as a function of PFAS chain length, at packing densities of 1.42 nm^–2^ (red) and 4.65 nm^–2^ (blue).

As shown in Figure 2, the MD-simulated water contact angles range from 90° to 130°, with clear dependencies on both chain length and packing density (*d*_p_). At low *d*_p_ (1.42 nm^–2^), the contact angle increases approximately linearly with chain length, reflecting improved surface coverage by fluorinated chains and reduced exposure of polar head groups. For head groups such as −CHO and −COOH, this leads to an increase of ∼20° (from ∼100° to ∼120°). In contrast, PFAS with −OH head groups show a more modest increase, suggesting that hydrogen-bonding interactions with water partially offset the effect of increasing fluorinated chain length. The most pronounced sensitivity is observed for −SO_3_H-terminated PFAS, where the contact angle increases by nearly 30°. This stronger dependence arises because, at short chain lengths, the highly polar −SO_3_H group remains more exposed to the interface, enhancing water affinity; increasing chain length progressively shields this group, leading to a sharper rise in hydrophobicity.

At high *d*_p_ (4.65 nm^–2^), the contact angle becomes largely insensitive to chain length for −CHO, −COOH, and −OH head groups, remaining within 110°–120°. This behavior indicates that dense packing promotes a more uniform fluorinated surface, effectively masking head group effects. However, the shortest −SO_3_H PFAS still exhibits relatively low water repellency, as tight packing cannot fully compensate for the strong polarity of this group when the chain is short. With increasing chain length, even −SO_3_H-terminated PFAS achieve contact angles above 110°, consistent with improved shielding of the head group.

These trends are in qualitative agreement with experimental measurements on idealized poly­(fluoroalkyl acrylate) (PFA) thin films, supporting the validity of the modeling approach.^16^ For instance, PFAS with a chain length of 2 exhibit a reported contact angle of 111°, which lies between the simulated values at low and high *d*_p_. Longer chains (lengths 4, 6, and 8) show similar contact angles in the range of 122°–126°, more than 10° higher than those with chain length 2. Comparing head group effects, −OH-terminated PFAS generally yield higher contact angles, whereas −SO_3_H-terminated PFAS exhibit reduced repellency at short chain lengths due to the presence of multiple oxygen atoms that increase polarity. Notably, −SO_3_H systems show a consistent increase in contact angle with chain length at both packing densities, underscoring the importance of head group shielding.

The contact angles of *n*-decane (Figure 3) fall within ∼60° to 100°, approximately 30° lower than the corresponding water contact angles due to the lower surface tension of decane. The dependence on chain length and packing density follows trends similar to those observed for water. At low *d*_p_, *n*-decane contact angles increase with chain length as fluorinated segments increasingly dominate the interface. At high *d*_p_, the contact angle becomes largely insensitive to chain length for most head groups, again reflecting the formation of a densely packed, low-energy surface.

A notable deviation is observed for −SO_3_H terminated PFAS, particularly in Figure 3d. The shortest PFAS exhibits a significantly lower *n*-decane contact angle (∼63°). Rather than direct interactions between the polar −SO_3_H group and nonpolar *n*-decane, we attribute this behavior to incomplete shielding of the surface by the short chains. Reduced CF_*x*_ surface coverage increases the effective surface energy through greater exposure of the head group region and potentially the underlying Nylon substrate. Because *n*-decane has a relatively low surface tension, it can more readily conform to this heterogeneous interface and access intermolecular free volume within the PFAS layer. As chain length increases, improved CF_*x*_ coverage produces a more uniform low energy surface, resulting in higher *n*-decane contact angles.

### 3.2 Contact Angle Prediction via Iterative Error-Driven Refinement

The systematic trends observed for single-chain PFAS establish a physically meaningful baseline linking PFAS molecular structure and packing density to water and oil repellency. However, single-chain PFAS represent only a small subset of the broader PFAS chemical space, which also includes numerous branched isomers whose wetting behavior may differ substantially despite having the same chain length. Extending these structure–property relationships to the full PFAS configurational space through direct MD simulations alone would be computationally prohibitive, motivating the development of a predictive and data-efficient modeling strategy.

In addition to linear PFAS, the configurational space encompasses branched PFAS with multiple isomers for a given chain length. For chain lengths from 1 to 7, including both linear and branched structures, the number of isomers increases rapidly with chain length, as summarized in Table 2. Considering all PFAS up to chain length 7, there are 58 unique molecules for each head group. Simulating all PFAS molecules across five surface packing densities (*d*_p_) and two liquid droplets (water and grade-6 oil) would require a total of 2320 MD simulations, rendering exhaustive sampling computationally infeasible. A machine learning approach with error-driven refinement was therefore developed to construct a comprehensive database efficiently.

**Table 2:** Number of Isomers for PFAS with Identical Head Groups and Chain Lengths from 1 to 7

PFAS of chain length	Number of isomers
1	1
2	1
3	2
4	4
5	7
6	14
7	29

The overall workflow is illustrated in Figure 4 and follows an iterative error-driven refinement strategy in which the training data set is iteratively and adaptively expanded based on model performance. The initial model was trained using the available linear PFAS data set (Section 3.1) and subsequently evaluated on branched PFAS structures representing a previously unexplored chemical space. The MD-derived contact angles of these branched PFAS structures provided feedback on model limitations, and only structures that contained new information by exhibiting significant prediction deviations (>3°) were incorporated into subsequent training iterations. Random forest algorithm (implemented in the scikit-learn Python package) models were trained separately for water and oil contact angles. PFAS molecules were represented using SMILES strings and encoded as Morgan fingerprints with a radius of 4 and a bit size of 2048, generated using RDKit. For each training run, the data set was randomly split into 80% training and 20% test sets. The resulting models achieved mean absolute errors (MAEs) below 2° for the training set and below 4° for the test set for both water and oil contact angles. This error is smaller than the standard error of the derived contact angles from MD simulation.

**Figure 4:**
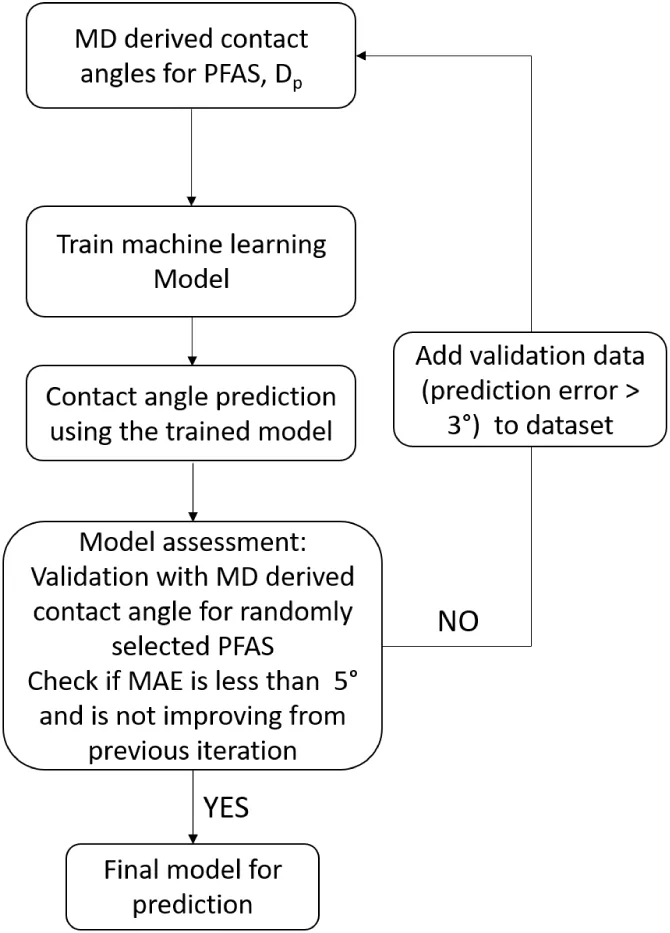
Workflow of the iterative error-driven refinement strategy used for predicting contact angles of water and oil droplet on PFAS coated Nylon.

To evaluate the applicability of the model beyond the single-chain PFAS domain, validation was conducted on branched PFAS compounds that were not included in the training data set. One branched PFAS was randomly selected for each chain length from 3 to 7, each head group, and each packing density, resulting in a validation set of 100 data points that uniformly spans four head groups, five chain lengths with isomeric structures, and five packing densities. MD simulations were performed to obtain reference contact angles for this validation set. The model performance on branched PFAS was significantly reduced, yielding MAEs of 7° and 6° for water and decane contact angles, respectively (Figure 5), indicating limited generalizability of the initial model.

**Figure 5:**
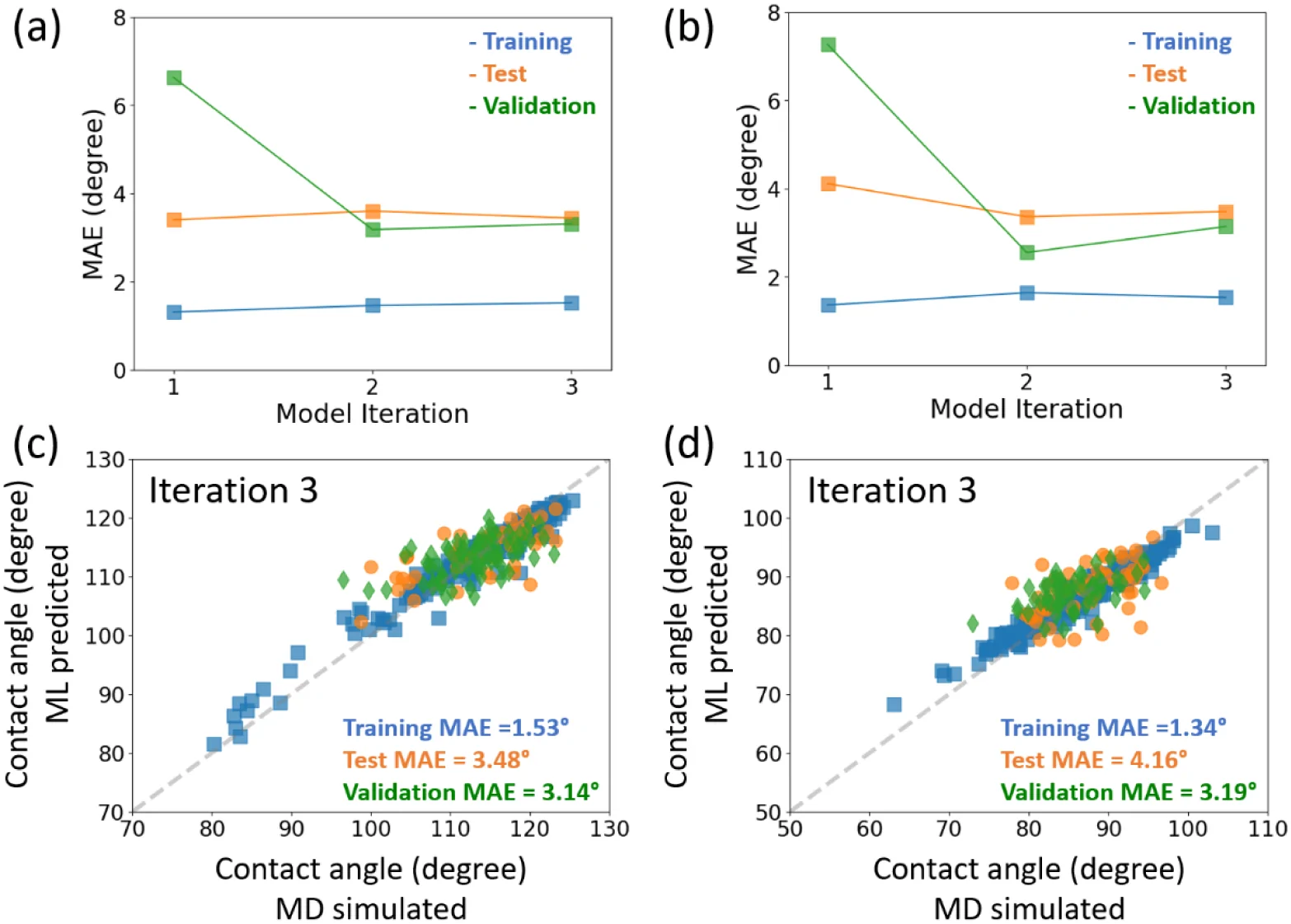
Mean absolute errors (MAEs) for training, testing, and validation data sets of the random forest model as a function of model iterations for the contact angle prediction of (a) water and (b) decane. Parity plot for the final model of (c) water and (d) decane.

To improve predictive accuracy, branched PFAS data points with prediction errors exceeding 3° were added to the training data set for the second iteration. The training and test MAEs remained comparable to those of the first iteration. A new validation set was then constructed by randomly selecting one previously unseen branched PFAS for each chain length (4–7), each head group, and each packing density. MD simulations were again performed to obtain ground-truth contact angles. As shown in Figure 5, the validation MAEs in the second iteration decreased to below 4° for both water and decane contact angles, demonstrating a substantial improvement in predictive performance.

To repeat the validation steps, validation data from the second iteration with prediction errors greater than 3° were incorporated into the training set, and a new validation set was generated following the same selection strategy. The resulting validation MAEs for both water and decane contact angles remained below 4°, comparable to those of the second iteration. Because the validation accuracy no longer improved substantially and the target accuracy had been achieved, the error-driven refinement loop was terminated. The final model is thus capable of accurately predicting water and decane contact angles for both single-chain and branched PFAS coatings across the considered range of molecular structures and surface packing densities.

### 3.3 Ensemble Behavior of PFAS Coatings in Hydrophobicity and Oleophobicity

After conducting error-driven refinement, comprehensive data sets describing the hydrophobicity and oleophobicity of PFAS coatings were generated for chain lengths ranging from 1 to 7, encompassing both linear and branched structures across a range of packing densities. This broad representation is important because, in practical applications, PFAS coatings are rarely composed of a single, well-defined molecular species. Instead, experimental formulations typically contain mixtures of compounds with diverse molecular structures.^47^ Even when coatings are nominally based on PFAS with a specific chain length, they generally include a combination of both linear and branched isomers.^48,49^

Information about the packing density of PFAS coatings on Nylon is generally unavailable. However, as shown in Section 3.1, packing density is a major factor influencing repellency for linear PFAS. The impact of packing density on repellency was analyzed for ensembles of PFAS considering linear and branched PFAS, grouped by head group, as shown in Figure 6a–d and Figure 6e–h for water and oil repellency, respectively.

**Figure 6:**
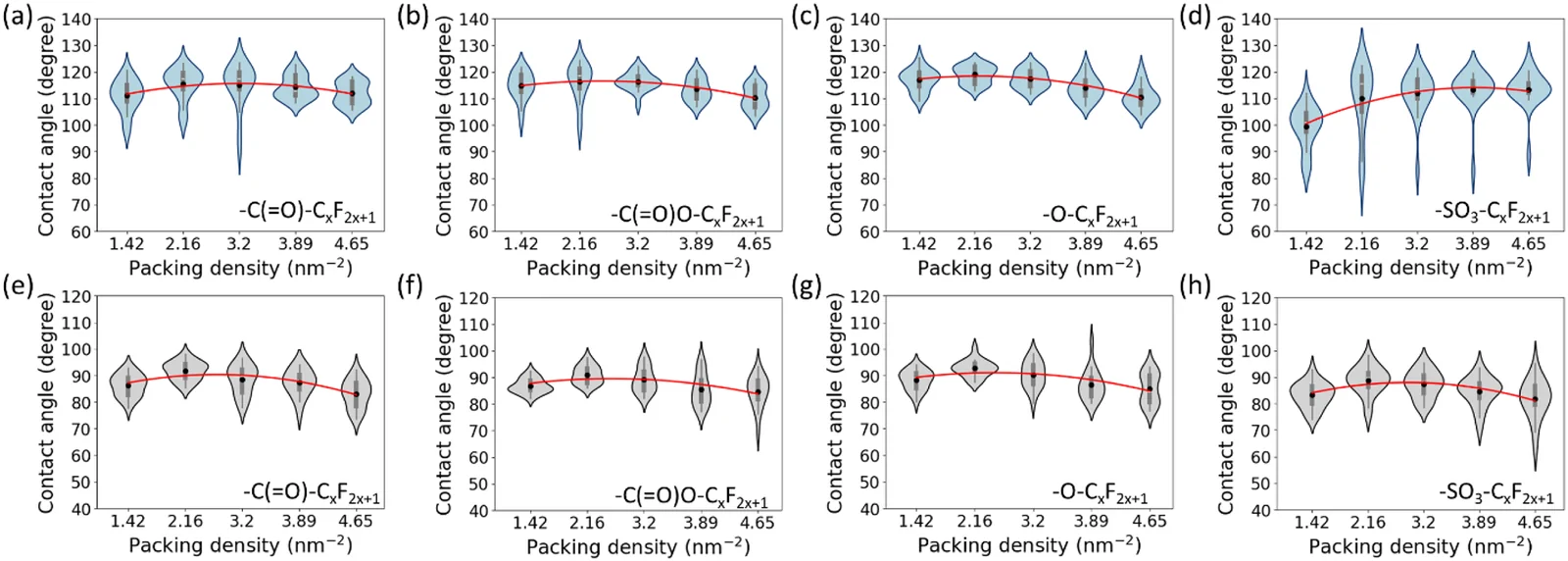
Contact angles of water droplets on Nylon surfaces coated by PFAS with head groups (a) −CHO, (b) −COOH, (c) −OH, and (d) −SO_3_H, and contact angles of oil droplets on Nylon surfaces coated by PFAS with head groups (e) −CHO, (f) −COOH, (g) −OH, and (h) −SO_3_H, shown as a function of PFAS packing density.

These results show that the highest mean water contact angles occur at intermediate packing densities. For PFAS with −COOH head groups, the maximum mean water contact angle is observed at a packing density of 3.2 nm^–2^ (Figure 6b). For PFAS containing −COOH or −OH groups, optimal repellency occurs at a packing density of 2.16 nm^–2^ (Figure 6f,g). For PFAS with −SO_3_H head groups, the highest oil repellency is observed at 2.16 nm^–2^, whereas water repellency shows a continuously increasing trend with *d*_p_ (Figure 6d). The range of contact angles at a given packing density is broader for PFAS with −SO_3_H, reflecting their greater sensitivity to chain length. A similar dependence on packing density is observed for oil repellency. Overall, the best repellency is consistently achieved at intermediate packing densities within the range considered.

The effects of chain length on water and oil repellency are shown in Figure 7. For water repellency, an increasing trend with chain length is observed for PFAS with −CHO and −SO_3_H head groups (Figure 7a, d). PFAS with shorter chain lengths exhibit a wider range of contact angles, reflecting their greater sensitivity to packing density. In contrast, oil repellency is less sensitive to chain length than water repellency. For PFAS with −CHO, −OH, and −COOH head groups, decane contact angles generally fall within a narrow range of approximately 80 to100° (Figure 7e–g). Notably, only short-chain PFAS (C1–C3) with SO_3_H head groups show a substantial fraction of contact angles below 80° (Figure 7h). In comparison between water and decane, the contact angles of water are about 10 to 20° higher than decane, consistent with the higher surface tension of water.^7,50^

**Figure 7:**
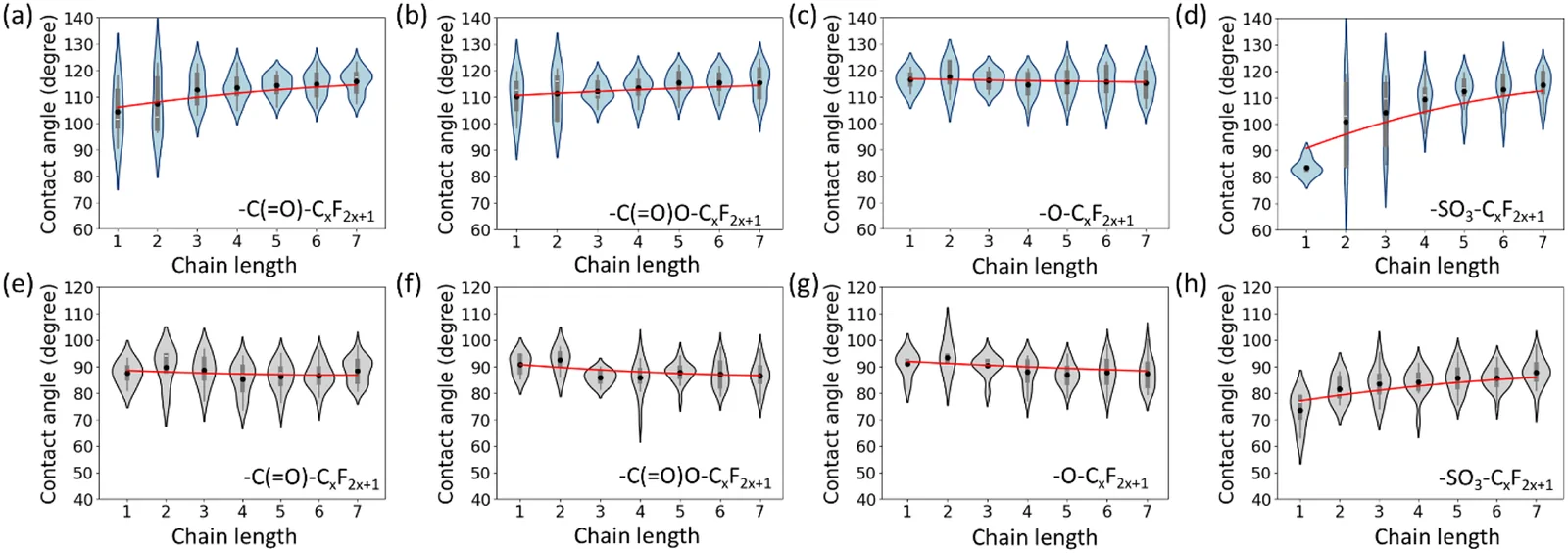
Contact angles of water droplets on Nylon surfaces coated by PFAS with head groups (a) −CHO, (b) −COOH, (c) −OH, and (d) −SO_3_H as a function of PFAS chain length. Contact angles of decane droplets on Nylon surfaces coated by PFAS with head groups (e) −CHO, (f) −COOH, (g) −OH, and (h) −SO_3_H as a function of PFAS chain length.

### 3.4 Assessment and Experimental Comparison of MD-Derived Contact Angles

The MD-derived contact angles were assessed using three complementary comparisons: (i) published MD simulation and experimental data for fluorinated surfaces,^16,18,51^ (ii) experimental measurements on commercially PFAS-treated Nylon fabric, and (iii) a complementary experimental assessment using ODA-coated Nylon sheets. Because the present MD model represents an idealized flat Nylon(010) surface, contact angles measured on textile substrates include additional contributions from fiber morphology, surface roughness, porosity, and other multiscale structural effects. Consequently, the PFAS-treated fabric measurements are interpreted as experimental comparisons for practical PFAS-coated Nylon systems rather than direct one-to-one validation of the intrinsic equilibrium contact angles predicted by the MD model. In contrast, the ODA-coated Nylon sheet provides a relatively smooth and well-defined substrate that more closely resembles the geometry represented in the simulations and therefore provides a more geometrically comparable system for an experimental assessment of the MD methodology.

The simulated water contact angles for the PFAS systems generally range from approximately 100° to 130°. These values capture the experimentally observed chain-length dependence and fall within the range reported for water contact angles on fluorinated planar surfaces.^16^ Yadav et al. reported experimental water contact angles of approximately 126–134° for perfluoroalkyl acrylate thin films with fluorinated chain lengths from C4 to C10 and demonstrated good agreement between MD simulations and experiments.^18^ Their simulations predicted microscopic contact angles up to approximately 132°, and, except for the shortest-chain PFA-C2 system, the relative deviations between simulation and experiment were below 5%. The present simulations capture the same overall dependence on fluorinated chain length across the different PFAS head group chemistries (−CHO, −COOH, −OH, and −SO_3_H), where the water contact angle increases rapidly with chain length before approaching a plateau (Figure 7). Although the absolute contact angles vary with head group chemistry, the experimentally observed chain length dependence is consistently captured. These results demonstrate that water contact angles in the range of 120–130° are consistent with previous experimental observations for fluorinated planar surfaces and provide a relevant benchmark for the magnitude and trends predicted by the present simulations.

The commercially finished C6 PFAS-treated Nylon fabric exhibited a measured water contact angle of 134±2° (Table 3), which is comparable to the upper range of the simulated values. Because this measurement was performed on a textile substrate, the observed contact angle includes morphology-dependent contributions^47^ that are not represented in the present molecular model. We therefore report this measurement only as a practical comparison for a PFAS-treated Nylon system and do not use it as quantitative validation of the intrinsic equilibrium contact angle predicted by the planar MD model.

**Table 3:** Experimental Water Contact Angles on PFAS Coatings

Liquid	Chemistry	Substrate	Contact angle	Source
Water	C6 PFAS	Nylon	134°±2°	This work
Water	C2 PFA	Carbon	98° ± 4°	ref. 16, 18
Water	C4 PFA	Carbon	126° ± 3°	ref. 16,18 ^,^
Water	C6 PFA	Carbon	129° ± 1°	ref. 16,18
Water	C8 PFA	Carbon	134° ± 2°	ref. 16,18
Water	C10 PFA	Carbon	132° ± 1°	ref. 16, 18
Grade 6 oil	C6 PFAS	Nylon	84°±3°	This work
Grade 6 oil	PFPE	Fabric	98° ± 4°	ref. 51

To provide a more geometrically comparable experimental assessment of the MD methodology, we additionally examined ODA-coated Nylon-6,6 sheets (Table I in the SI). ODA is chemically distinct from PFAS and therefore provides an independent benchmark of the droplet-simulation workflow on a comparatively planar Nylon substrate. As shown in Table 4, the simulated and experimental water contact angles are 144° and 137 ± 2°, respectively, corresponding to a difference of 7°. This comparison provides additional experimental evidence for the MD workflow on a Nylon-based planar substrate.

**Table 4:** Experimental and Simulated Water and Oil Contact Angles on ODA Coating

Liquid	Chemistry	Substrate	Contact angle	Source	Method
Water	ODA	Nylon	137° ± 2°	This work	Exp.
Water	ODA	Nylon	144°	This work	MD
Grade 6 oil	ODA	Nylon	77° ± 3°	This work	Exp.
Grade 6 oil (*n*-decane)	ODA	Nylon	88°	This work	MD

A similar assessment strategy was adopted for the simulated *n*-decane contact angles. Because grade 6 oil in the AATCC 118 hydrocarbon resistance test corresponds to *n*-decane, the measured grade 6 oil contact angles are reported as practical comparisons; because these measurements were obtained on textile substrates, they are not used as direct quantitative validation of the planar MD results. The commercially finished C6 PFAS-treated Nylon fabric exhibited a grade 6 oil contact angle of 84°±3° (Table 3). As discussed above, this measurement was performed on a textile substrate and therefore serves as an experimental comparison for a practical PFAS-coated Nylon system rather than a direct one-to-one validation of the intrinsic equilibrium contact angle predicted by the idealized flat-surface MD model.

Previous studies have demonstrated that oil contact angles depend strongly on surface morphology in addition to surface chemistry. For example, Golovin et al. distinguished the Young’s contact angle on smooth perfluoropolyether­(PFPE)-coated surfaces from the apparent contact angles on fibrous and hierarchically structured fabrics.^51^ While the smooth PFPE surface exhibited Young’s contact angle of 73° for n-hexadecane (Grade 3 AATCC TM118), the corresponding PFPE-treated fabric exhibited apparent contact angles of 118° for n-hexadecane on fibers, 150° ± 4° for n-hexadecane and 98° ± 6° for n-decane (Grade 6 AATCC TM118) on hierarchically structured fabricsane.^51^ These observations highlight that substrate morphology can substantially enhance apparent oil repellency and should be considered when comparing contact angles across different systems.

To further assess the MD droplet-simulation methodology on a comparatively planar Nylon substrate, we performed an experimental assessment using ODA-coated Nylon sheets, which provide a relatively smooth and well-defined substrate more closely matching the modeled geometry. As shown in Table 4, the simulated and experimental *n*-decane contact angles are 88° and 77 ± 3°, respectively, corresponding to a difference of 11°. Together with the corresponding 7° difference for water, these results provide a complementary experimental assessment of the MD workflow on a chemically distinct coating system and establish the approximate level of quantitative agreement obtained with the present methodology.

Published studies further demonstrate the importance of distinguishing intrinsic equilibrium contact angles on smooth surfaces from apparent contact angles measured on structured textile substrates. The oil contact angles were found much smaller for PFAS coatings on silicon substrates, where the value can be measured as low as 55° *n*-decane (Grade 6 AATCC TM118) droplet for PFPE treated silicon wafer, as conducted by Golovin et al.^52^ In another work, Golovin et al.^51^ reported a Young contact angle of 73 ± 1° for n-hexadecane (Grade 3 AATCC TM118) on a smooth PFPE-coated silicon wafer, whereas the corresponding apparent contact angles increased to 118 ± 1° on PFPE-treated fabrics and 150 ± 4° on hierarchically structured PFPE-treated fabrics. The same hierarchically structured fabric exhibited an apparent *n*-decane contact angle of 98 ± 4°. These results clearly demonstrate that surface morphology can substantially increase the apparent oil contact angle and therefore should be considered when comparing contact angles measured on different physical systems. We acknowledge that our MD simulation derived contact angles are on an ideal PFAS coating attached to a planar surface of Nylon substrate, which has limited similarity with real PFAS coating systems on substrates such as silicon or fabric with more complex surface structures such as their surface morphology, weave architecture, surface roughness, porosity, coating uniformity, liquid penetration, and multiscale capillary effects.^43^

Taken together, the literature comparisons and the ODA-coated Nylon measurements provide useful benchmarks for assessing the present MD methodology while also defining its limitations. The simulated water contact angles capture the experimentally observed chain-length dependence and fall within the range reported for fluorinated planar surfaces.^16,18,51^ For *n*-decane, the ODA-coated Nylon comparison indicates that the methodology captures the qualitative wetting behavior and overall magnitude of the contact angles but retains quantitative uncertainty for low-surface-tension liquids. Measurements on structured textile substrates are therefore used only as practical comparisons and not as quantitative validation of the planar MD predictions.

## 4 Conclusions

In this study, a machine learning-driven molecular dynamics framework was developed to predict the intrinsic water and oil wettability behavior of PFAS coatings over Nylon toward both water and oil. By systematically sampling head group chemistries (−CHO, −COOH, −OH, and −SO_3_H), chain lengths from 1 to 7, and surface packing densities ranging from 1.42 to 4.65 nm^–2^, the resulting model achieved high predictive accuracy, with mean absolute errors below 3° for contact angles. For linear PFAS, water contact angles increased linearly with chain length at low packing densities but became largely insensitive to chain length at high packing densities, except for −SO_3_H head groups, while oil contact angles exhibited weaker overall chain-length dependence, due to weaker interaction with PFAS head groups and Nylon substrate.

The framework was further extended to branched PFAS isomers, where error-driven refinement reduced prediction errors to below 5° within three iterations, enabling the construction of a comprehensive database capturing hydrophobicity and oleophobicity across both linear and branched chemistries. Ensemble analysis revealed that maximum mean contact angles occur at intermediate packing densities, rather than at the highest packing densities, for both liquids. Chain length positively correlated with contact angle for −CHO and −SO_3_H head groups, whereas −COOH and −OH showed minimal sensitivity. Consistent with experimental observations, oil contact angles were approximately 20° lower than water contact angles under identical conditions, reflecting the lower surface tension of oils.

Overall, this study provides mechanistic insight into PFAS-based intrinsic water and oil wettability, and establishes a robust, data-driven foundation for understanding structure–property relationships in liquid-repellent coatings. Importantly, the error-driven refinement approach and MD-derived insights developed here are readily transferable to the discovery of sustainable, non-PFAS alternatives. Future efforts integrating high-throughput MD simulations with generative artificial intelligence will enable accelerated screening and design of environmentally benign non-PFAS coating materials with tunable water and oil wettability performance and long-term durability.

## Supplementary Material





## Data Availability

The LAMMPS molecular dynamics input and data files, contact angle derivation code, and machine learning code used for model development are available in the Supplementary Data file. Correspondence and requests for materials should be addressed to F.C.

## Notes

Supplementary data (ZIP)
Contact angle calculation methods, effects of water and *n*-decane droplet sizes, molecular dynamics simulation time, and sensitivity analyses of density thresholds and spatial binning (PDF)
