# Supplementary material for: Machine Learning with Iterative Error-Driven Refinement for Predicting PFAS-Coated Surface Wettability
Source: ACS Mater Au. 2026 Oct 24;6(6):1673–83. doi: 10.1021/acsmaterialsau.6c00228 (PMC13760661; doi:10.1021/acsmaterialsau.6c00228)
Supplement: Supplementary file 1 [file mg6c00228_si_002.pdf]

Supporting Information

Machine Learning with Iterative Error-Driven Refinement for Predicting PFAS-Coated Surface Wettability

Yilang Liu,[a] Jiaqi Yang,[b] Bahareh Rahimi,[c] Tejasri Remma,[c] Joshna Chittigori,[c] Jayant Kumar,[d] Gabriel Salierno,[e] Margaret A. Auerbach,[f] Ravi Mosurkal,[f] Ramaswamy Nagarajan,[c] and Fanglin Che[a,b*]

[a]Department of Chemical Engineering, University of Massachusetts Lowell, Lowell, MA 01854, USA

[b]Department of Chemical Engineering, Worcester Polytechnic Institute, Worcester, MA 01609, USA

[c]Department of Plastics Engineering, University of Massachusetts Lowell, Lowell, MA 01854, USA

[d]Department of Physics, University of Massachusetts Lowell, Lowell, MA 01854, USA

[e]Toxics Use Reduction Institute (TURI), University of Massachusetts Lowell, Lowell, MA 01854, USA

[f]Soldier Protection Directorate, US Army DEVCOM Soldier Center, Natick, MA 01760, USA

*Corresponding author: fche@wpi.edu

1. Contact angle derivation

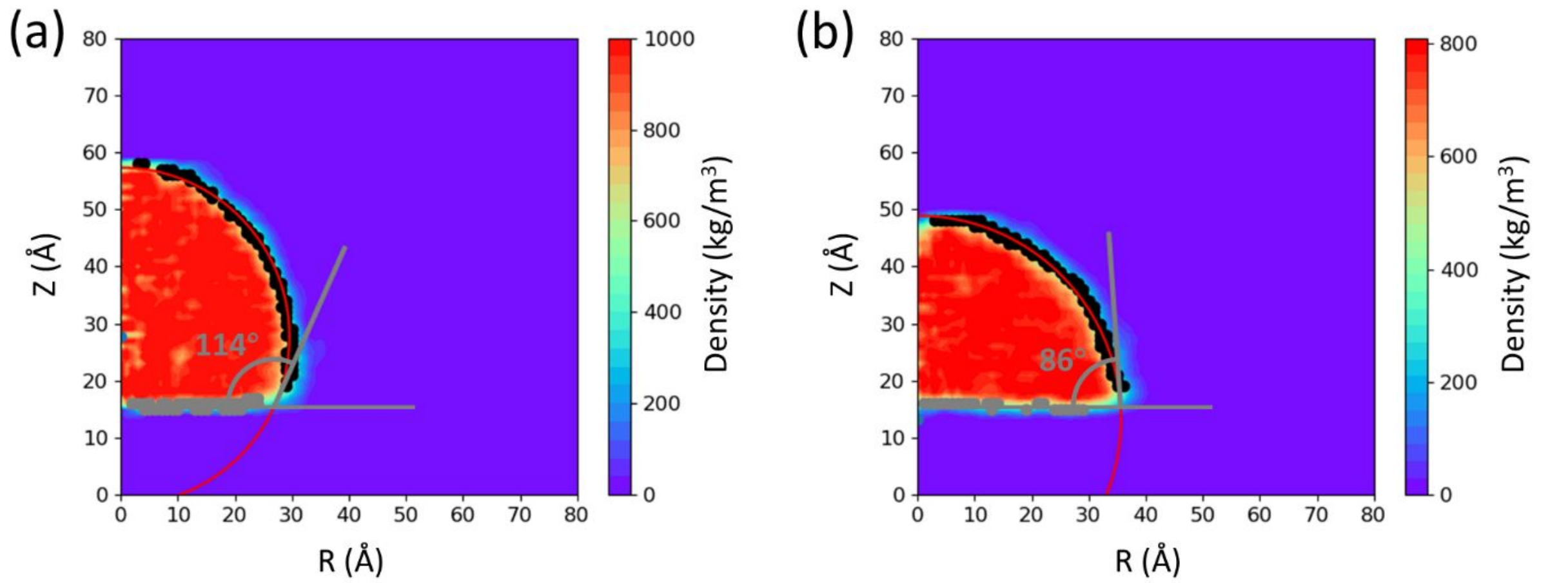

Figure S1. Density contour plot of (a) water droplet at 500 picosecond and (b) n-decane droplet at 1000 picosecond on $CF_3(CF_2)_3COOH$ coated Nylon at packing density of 4.65 nm^{-2}. Black dots denote the coordinates at the interface of liquid and air, which are used to fit to a circle as represented by the red curve. Grey dots denote the coordinates at the interface of liquid and PFAS coating surface. The contact angles are calculated at the intersection of the red circle and coating surface line.

2. Determination of molecular dynamics (MD) simulation time and liquid size

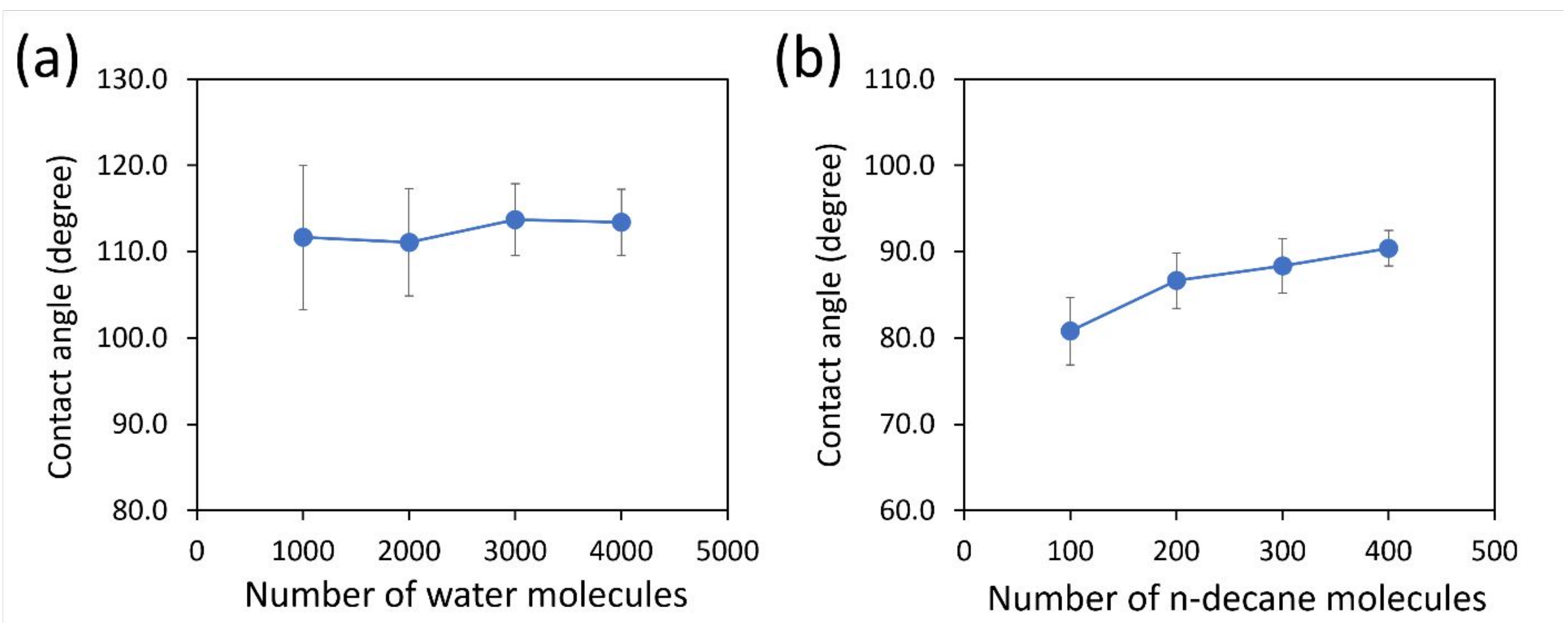

Figure S2. Contact angle with standard error as a function of number of droplet size for (a) water and (b) n-decane on $CF_3(CF_2)_3COOH$ coated Nylon at packing density of 4.65 nm^{-2}.

Table S1. Contact angle of water droplet of different sizes on $CF_3(CF_2)_3COOH$ coated Nylon at packing density of 4.65 nm^{-2}.

Number of water molecules	Contact angle (degree)	Standard error (degree)
1000	111.67	8.37
2000	111.07	6.19
3000	113.70	4.18
4000	113.39	3.83

Table S2. Contact angle for n-decane droplet of different sizes on $CF_3(CF_2)_3COOH$ coated Nylon at packing density of 4.65 nm^{-2}.

Number of n-decane molecules	Contact angle (degree)	Standard error (degree)
100	80.79	3.88
200	86.65	3.20
300	88.35	3.15
400	90.39	2.06

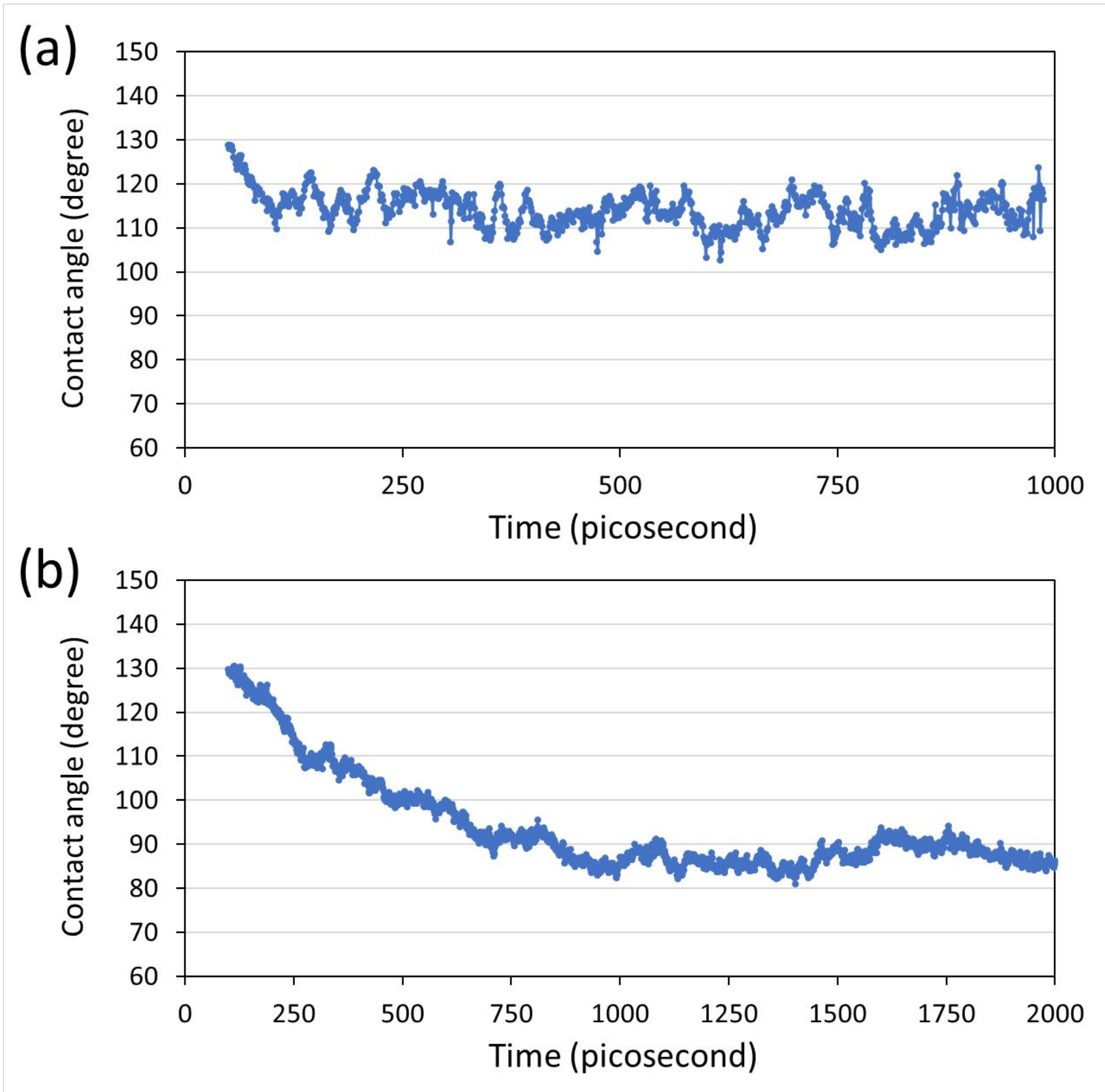

Figure S3. Molecular dynamics derived contact angle as a function of simulation time for (a) water and (b) oil droplets on $CF_3(CF_2)_3COOH$ coated Nylon at packing density of 4.65 nm^{-2}. Water contact angle becomes stable after 200 picoseconds and n-decane contact angle becomes stable after 750 picoseconds.

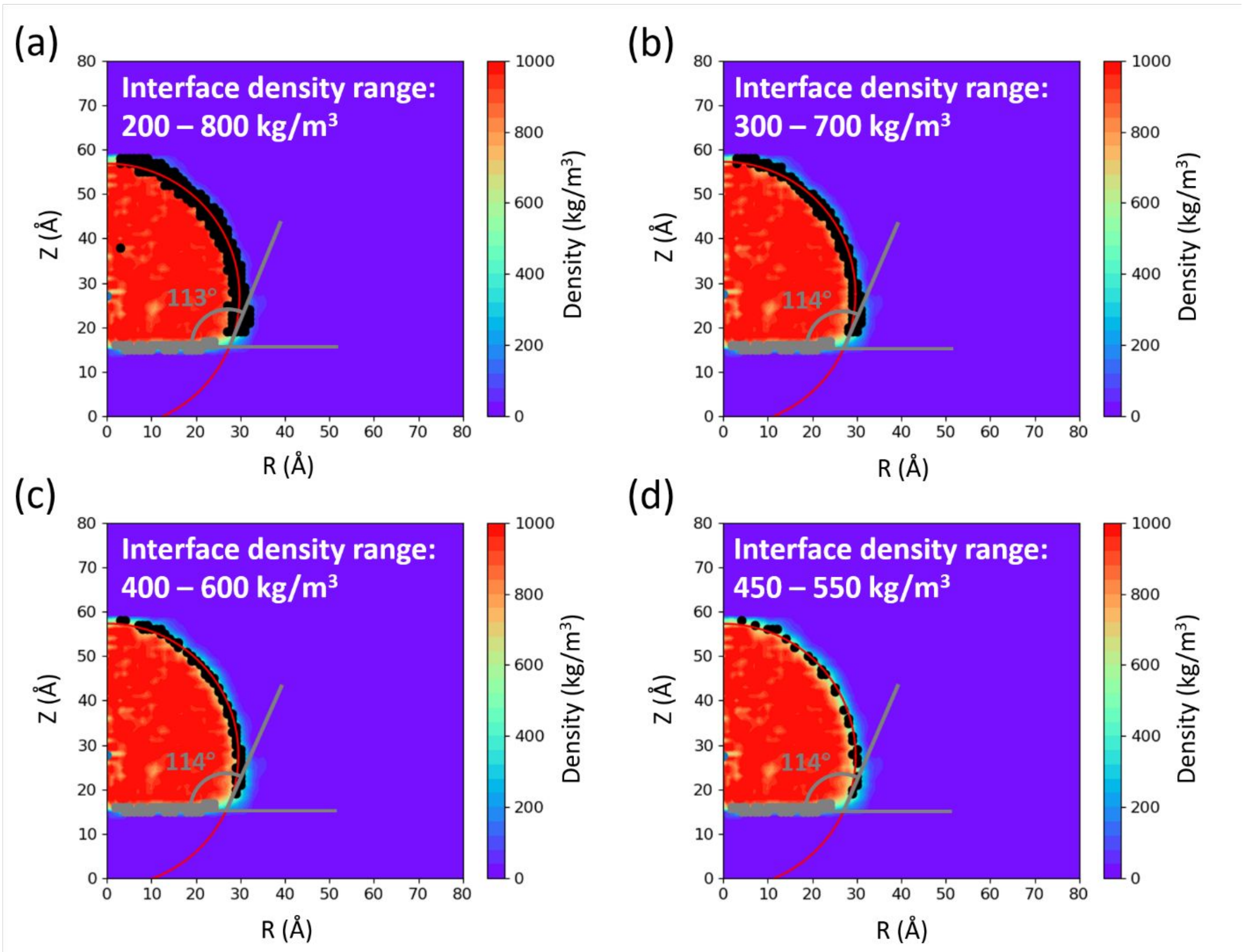

Figure S4. Density contour plots of water droplet at 500 picoseconds on $CF_3(CF_2)_3COOH$ coated Nylon at packing density of 4.65 nm^{-2}, fitted using different water density ranges representing water-gas interface: (a) 200-800 kg/m^3, (b) 300-700 kg/m^3, (c) 400-600 kg/m^3, and (d) 450-550 kg/m^3. Black dots denote the coordinates at the interface of liquid and air, which are used to fit to a circle as represented by the red curve. Grey dots denote the coordinates at the interface of liquid and PFAS coating surface. The contact angles are calculated at the intersection of the red circle and coating surface line.

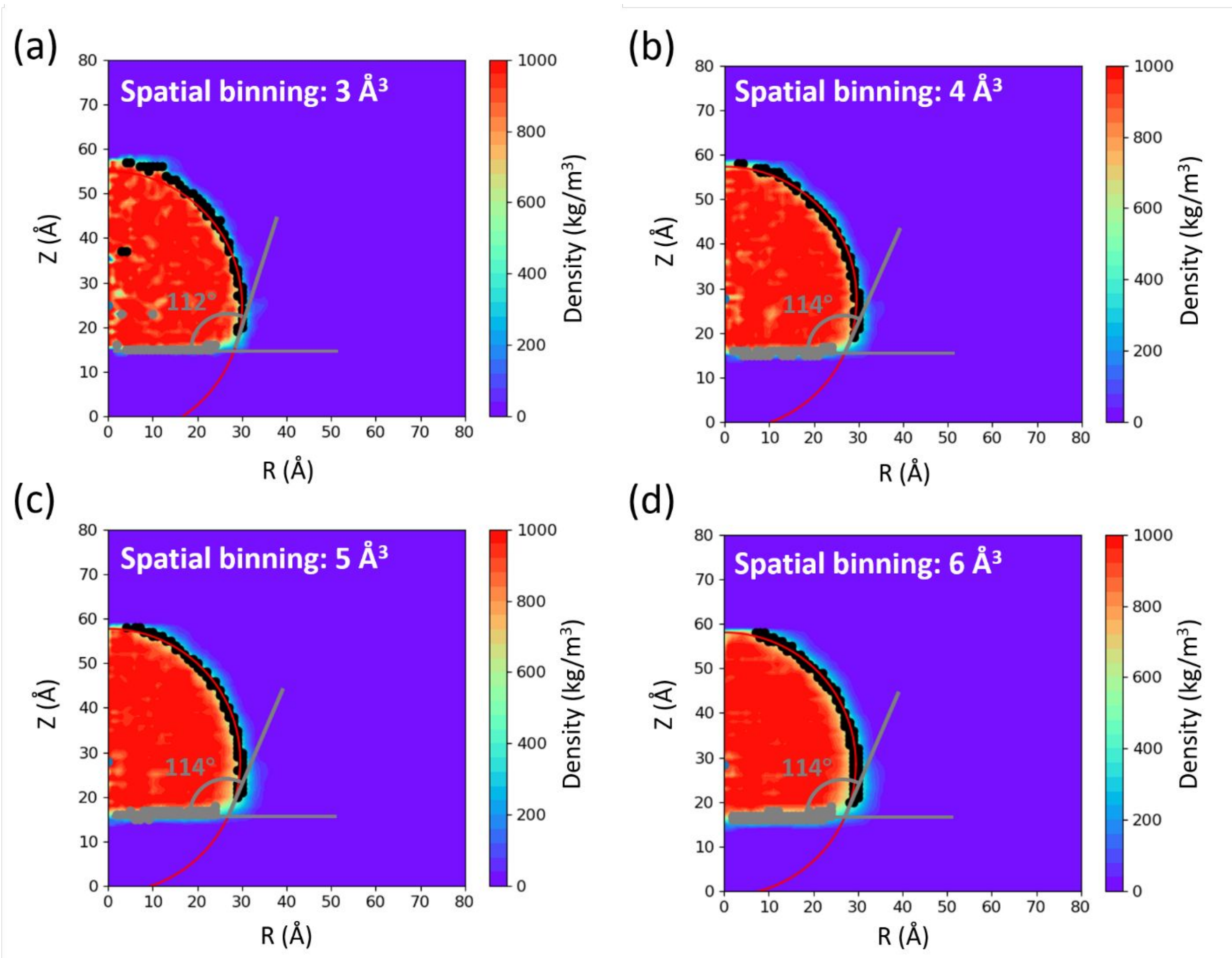

Figure S5. Density contour plots of water droplet at 500 picoseconds on $CF_3(CF_2)_3COOH$ coated Nylon at packing density of 4.65 nm^{-2}, fitted using different spatial binning: (a) 3 $Å^3$, (b) 4 $Å^3$, (c) 5 $Å^3$, and (d) 6 $Å^3$. Black dots denote the coordinates at the interface of liquid and air, which are used to fit to a circle as represented by the red curve. Grey dots denote the coordinates at the interface of liquid and PFAS coating surface. The contact angles are calculated at the intersection of the red circle and coating surface line.

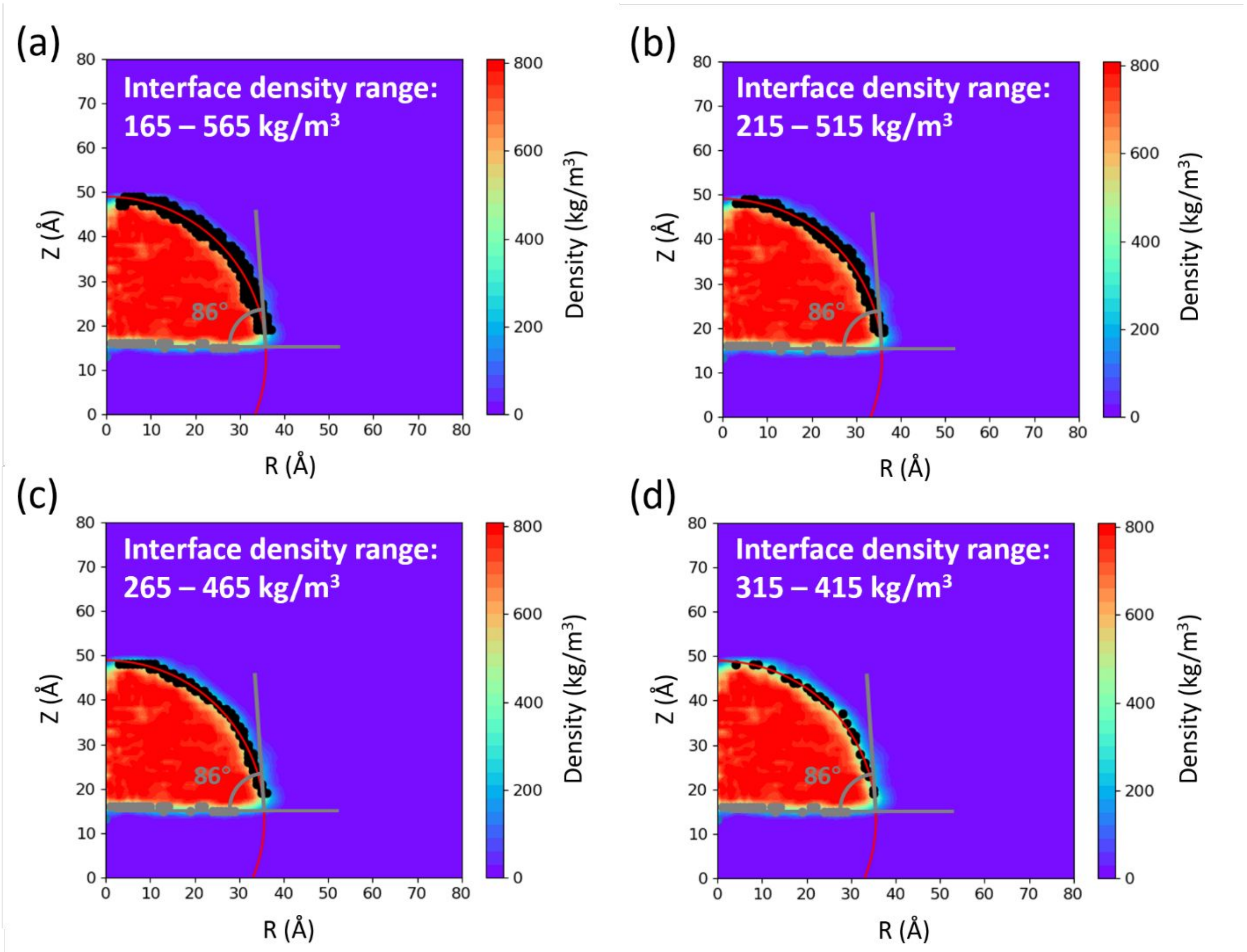

Figure S6. Density contour plots of n-decane droplet at 1000 picoseconds on $CF_3(CF_2)_3COOH$ coated Nylon at packing density of 4.65 nm^{-2}, fitted using different oil density ranges representing oil-gas interface: (a) 165-565 kg/m^3, (b) 215-515 kg/m^3, (c) 265-465 kg/m^3, and (d) 315-415 kg/m^3. Black dots denote the coordinates at the interface of liquid and air, which are used to fit to a circle as represented by the red curve. Grey dots denote the coordinates at the interface of liquid and PFAS coating surface. The contact angles are calculated at the intersection of the red circle and coating surface line.

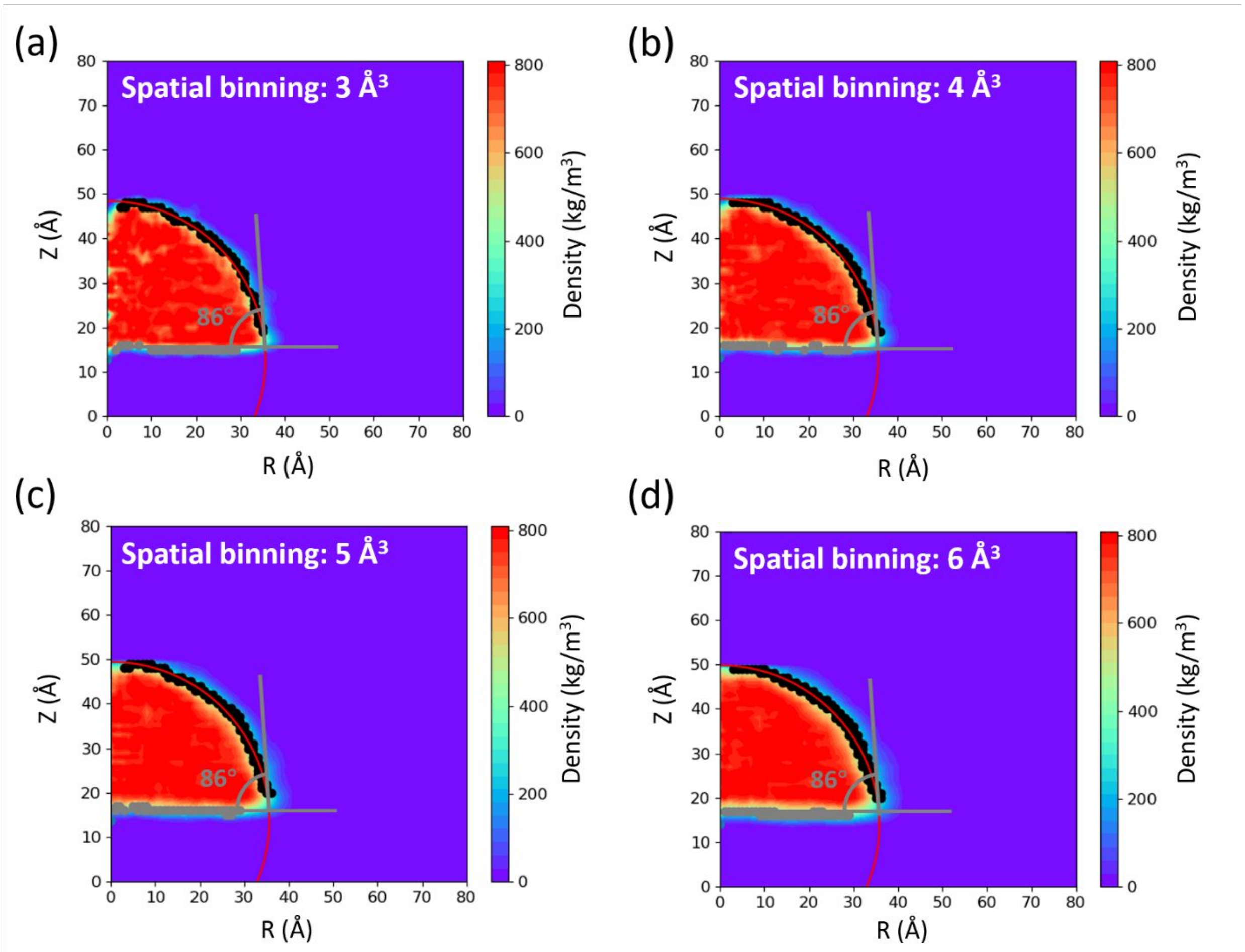

Figure S7. Density contour plots of n-decane droplet at 1000 picoseconds on $CF_3(CF_2)_3COOH$ coated Nylon at packing density of 4.65 nm^{-2}, fitted using different spatial binning: (a) 3 $Å^3$, (b) 4 $Å^3$, (c) 5 $Å^3$, and (d) 6 $Å^3$. Black dots denote the coordinates at the interface of liquid and air, which are used to fit to a circle as represented by the red curve. Grey dots denote the coordinates at the interface of liquid and PFAS coating surface. The contact angles are calculated at the intersection of the red circle and coating surface line.
